# Interface Rectification via Roll‐to‐Roll Dual‐Confinement Nanocoatings for High‐Temperature Polymer Capacitors

**DOI:** 10.1002/advs.78275

**Published:** 2026-10-14

**Authors:** Ning Guo, Marina Dabaghian, Jierui Zhou, Josh N. Bodin, Chong Xiang, Fei Wang, Antigoni Konstantinou, Dingxin Wei, Yifei Wang, Chao Wu, Riccardo Casalini, Bryan Huey, Priya Vashishta, Luyi Sun, Yang Cao

**Affiliations:** ^1^ Electrical Insulation Research Center Institute of Materials Science University of Connecticut Storrs Connecticut USA; ^2^ Department of Electrical and Computer Engineering University of Connecticut Storrs Connecticut USA; ^3^ Department of Chemical and Biomolecular Engineering University of Connecticut Storrs Connecticut USA; ^4^ Polymer Program Institute of Materials Science University of Connecticut Storrs Connecticut USA; ^5^ GE Vernova Advanced Research Center Niskayuna New York USA; ^6^ Department of Materials Science & Engineering University of Connecticut Storrs Connecticut USA; ^7^ Chemistry Division Naval Research Laboratory Washington DC USA; ^8^ School of Materials Engineering Purdue University West Lafayette Indiana USA; ^9^ Collaboratory for Advanced Computing and Simulations University of Southern California Los Angeles California USA

## Abstract

Polymer film capacitors are indispensable for energy storage and power conversion, yet their energy density and efficiency deteriorate rapidly at elevated temperatures. At temperatures relevant to wide‐bandgap semiconductor electronics (>150°C), heat‐resistant polymer films must be heavily derated to avoid premature aging due to intensive hot‐carrier injection. Inspired by multilayered barrier structures in nature, we developed a scalable roll‐to‐roll self‐assembled organic‐inorganic nanolaminate coating that significantly enhances the high‐temperature dielectric performance of polymer films. The nanolaminate architecture introduces dual confinement mechanisms: nanoconfinement of the polymer binder suppresses chain motion and enhances thermal stability, while anisotropic carrier relaxation within two‐dimensional nanoclay nanosheets impedes through‐plane hot‐carrier transport. Together, these effects synergistically establish an interface‐rectification mechanism that promotes in‐plane energy dissipation and suppresses carrier injection at electrode‐polymer interfaces. At 150°C, the coating significantly reduces the pre‐breakdown conduction current of polyetherimide (PEI), increases the breakdown strength from 450 to 501 MV/m, and improves the discharged energy density at 95% efficiency by 105%. Furthermore, roll‐to‐roll‐coated PEI capacitors exhibit a 45‐fold lifetime enhancement at 150°C and 180 MV/m—a design field previously sustained only by BOPP below 60°C. This work provides a scalable and technology‐ready strategy for high‐temperature, high‐density polymer capacitive energy storage.

## Graphical abstract

Dual confinement in self‐assembled nanocoatings regulates polymer dynamics and hot‐carrier transport, enabling an interface‐rectification strategy for high‐temperature polymer capacitive energy storage.

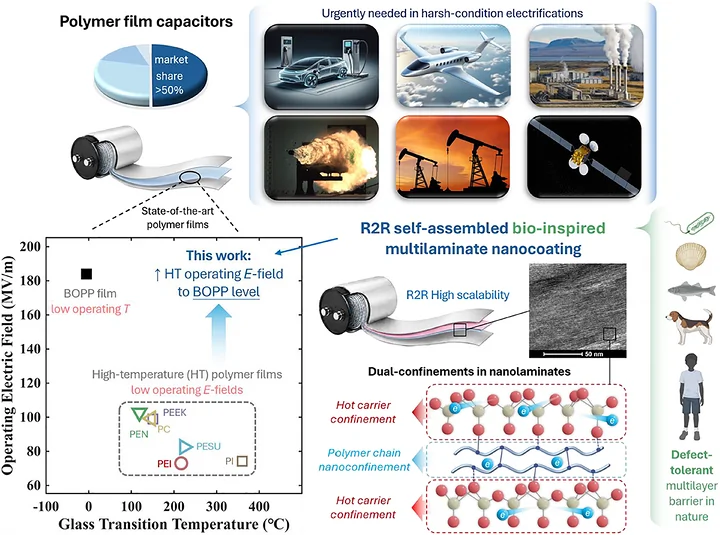

## 1 Introduction

Polymer film capacitors are critical components for energy storage and power conversion in modern high‐power‐density electronic and electrical systems [1, 2, 3]. With the rapid advancement of wide‐bandgap (WBG) semiconductors (e.g., SiC and GaN), many electrification systems are being driven toward increasingly harsh electrical (>200 MV/m) and thermal (>150°C) operating conditions [4, 5], exceeding the operating limits of existing polymer‐film capacitors [3, 6]. For instance, the state‐of‐the‐art commercial capacitor film, biaxially oriented polypropylene (BOPP), can sustain a high operating electric field of 184 MV/m but only at temperatures below 60°C [4]. In contrast, commercial heat‐resistant films can tolerate >150°C; however, owing to their aromatic backbone structures and consequently limited electronic bandgaps, their operating electric fields must be derated by 70∼80% to suppress conduction loss, resulting in energy densities far inferior to those of BOPP. Although promising large‑bandgap, heat‑resistant polymers have been developed through rational *bulk* molecular‐engineering design [5, 7, 8], their low manufacturing readiness presents a major barrier to near‐term practical adoption, as capacitors typically require kilometer‐scale, high‐quality films [9, 10]. Meanwhile, well‐established polymers such as Kapton (polyimide) and ULTEM (polyetherimide, PEI), despite decades of industrial development in thin film processing, have yet to be deployed in power capacitors owing to inherently low operating electric fields.

*Surface* engineering provides an effective and high‐technology‐readiness alternative [9]. Since hot‐carrier injection at the metal‐polymer interfaces accounts for the primary origin of coupled electro‐thermal‐mechanical‐chemical aging in polymer films [11, 12, 13, 14, 15], mature surface‐modification techniques can be directly applied to commercial heat‐resistant films to suppress hot‐carrier injection and enable high‐temperature operation at practical electric fields without derating. Existing approaches predominantly rely on homogenous surface coatings composed of a single functional material (e.g., Al_2_O_3_ or SiO_2_) [9, 13, 16, 17, 18, 19, 20, 21, 22], typically applied via cumbersome chemical or physical vapor depositions in vacuum. However, these methods suffer from limited scalability due to stringent processing requirements and, more critically, from intrinsic defect sensitivity: a single flaw or impurity can quickly evolve into a preferential conduction pathway. For kilometer‐long films in capacitor, such defects are statistically unavoidable and can induce dielectric failure [9]. These limitations highlight the urgent demands for a scalable and defect‐tolerant barrier coating strategy.

In fact, *nature* has long provided a highly effective and scalable paradigm for “defect‐free” barrier design. As illustrated in Figure 1a, protective shells, scales, and skin barriers—from bacteria [23] and mollusca [24] to fish [25] and mammal [26]—consistently adopt multilaminate architectures shaped by millions of years of natural selection. These structures are inherently *defect‐tolerant*: flaws in an individual layer are compensated by adjacent layers. More fundamentally, multilaminate architectures introduce abundant heterogeneous interfaces that disrupt through‐thickness transport of invasive species or energy, redirecting them into lateral dissipation (Figure 1a) [24, 26]. This principle has inspired a wide range of engineered barriers for impact resistance [24], thermal barrier [27], and acoustic attentuation [28]. In electrical insulation, the interfacial barrier within highly ordered lamellar structures can effectively impede hot carrier transport [29, 30, 31, 32]. High‐aspect‐ratio 2D fillers, when aligned in‐plane, similarly impede and redirect electron‐avalanche processes within polymer dielectrics, thereby enhancing dielectric strength [33, 34, 35, 36]. These advances in polymer bulk design provide a strong rationale for applying multilaminate structure into nanometric coatings. Uniquely, when such structure is reduced to the nanoscale, the constituent materials can experience pronounced confinement effect. Recent studies reveal that confinement can markedly restrict molecular motion [37, 38, 39, 40] and alter quasiparticle dynamics, including phonons [41, 42, 43], which are closely related to hot‐carrier relaxation processes [44, 45]. Quantum confinement in 2D materials also reduces the screening effect and enhances the electron‐phonon coupling, intrinsically improving breakdown strength [46]. Together, these insights indicate *bio‐inspired* multilaminate architecture a compelling route toward scalable, defect‐tolerant barrier coatings capable of suppressing carrier injection in polymer films.

**FIGURE 1:**
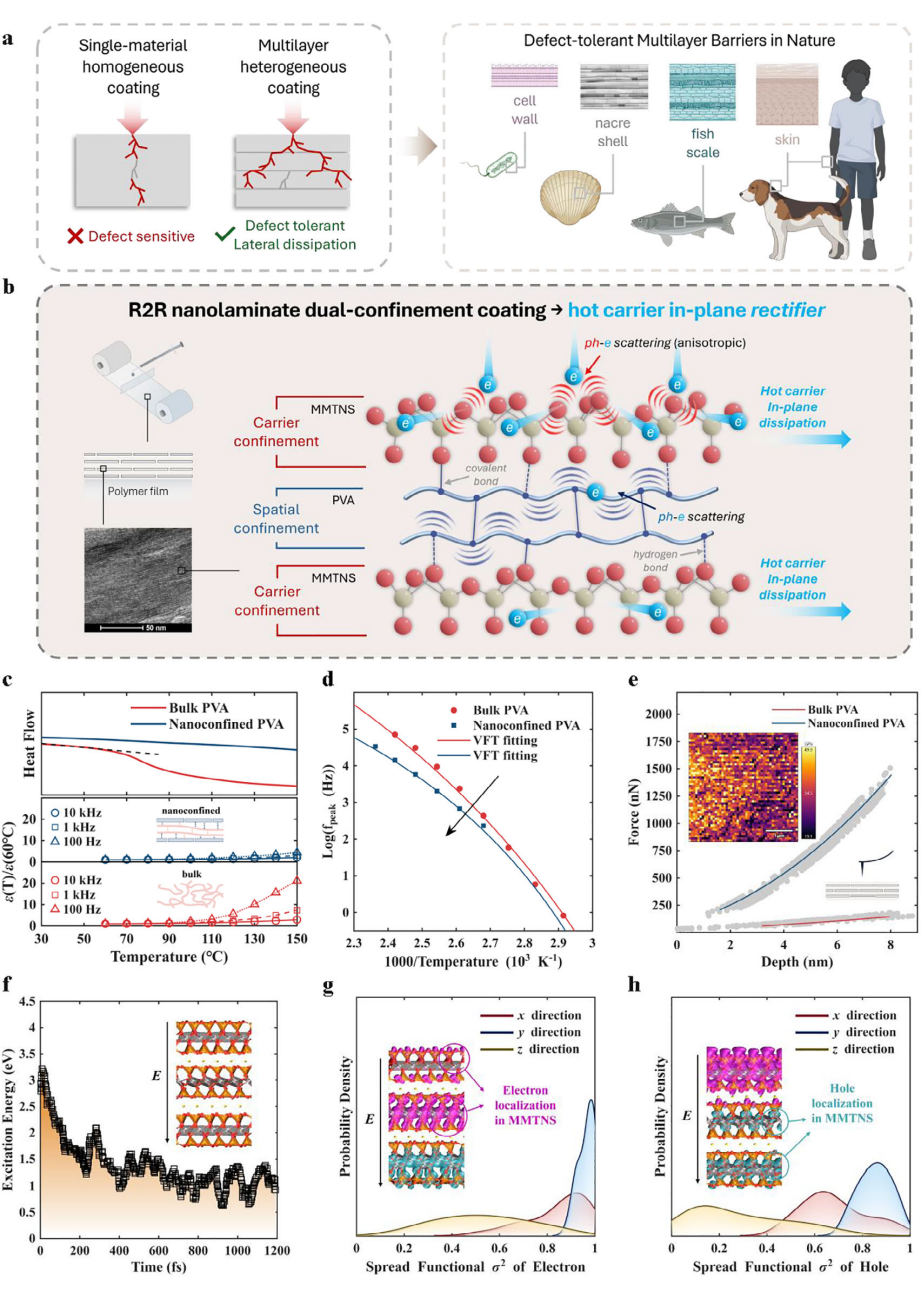
Dual confinements in the multilaminate nanocoating. (a) Schematic illustrating lateral redirection in multilaminate coatings and analogous multilaminate barrier structures found in nature; (b) Schematic of the roll‑to‑roll‑fabricated dual‑confinement nanometric in‐plane *rectifier*, together with a cross‑sectional TEM image of the multilaminate architecture; Enhanced electron scattering in nanoconfined organic layers, coupled with in‐plane carrier dissipation due to anisotropic localization in inorganic layers, synergistically suppresses through‐plane hot‐carrier transport and redirects carriers toward lateral pathways, thereby establishing a nanostructured rectifying architecture; (c) DSC measurements of neat PVA (bulk) and PVA/MMT (nanoconfined) coating layers, followed by the permittivity dispersion of PVA/MMT and neat PVA; (d) *α* relaxation peak frequency (*f*_max_) of neat PVA and PVA/MMT extracted from broadband dielectric spectroscopy; (e) Force‐depth curves of neat PVA and PVA/MMT; the up‐left inset shows the elastic modulus distribution of PVA/MMT, and the bottom‐left inset illustrates the AFM‐based nanoindentation schematic); (f) Rapid relaxation of excitation energy of hot carriers within 1200 fs; the inset shows NAQMD simulation model; (g, h) Probability density distribution of the normalized spread functional for g electrons and h holes within 1200 fs.

Here, we report a roll‐to‐roll self‐assembled multilaminate organic‐inorganic nanocoating that markedly enhance the high‐temperature insulation performance in heat‐resistant polymer films. A scalable roll‐to‐roll self‐assembly process integrates 2D montmorillonite (MMT) nanosheets with high‐molecular‐weight polyvinyl alcohol (PVA) directly onto polymer substrates, forming self‐*co*‐assembled coatings comprising hundreds of highly oriented organic/inorganic alternating nanolayers in a single pass. This massive layer redundancy confers effective *defect‐tolerance*, while the total thickness remains only a couple hundred nanometers. The resulting nanostructured coating imposes *dual confinements* within this surface barrier. PVA chains are nanoconfined between MMT nanosheets, forming an *immobilized* matrix with improved thermal stability and enhanced acoustic phonon scattering. Simultaneously, hot‐carriers undergo rapid *anisotropic* localization within MMT nanosheets, as revealed by non‐adiabatic quantum molecular dynamic (NAQMD) simulations, exhibiting suppressed out‐of‐plane transport and enhanced in‐plane dissipation. Together, these effects render the surface coating a thin, nanostructured high‐voltage (HV), in‐plane *rectifier* that synergistically inhibits hot carrier injection and transport into underlying polymer films. When applied to PEI film, the nanocoating markedly suppresses nonlinear conduction at 150°C (reducing hopping distance by 46%), increases breakdown strength from 450 to 501 MV/m, and enhances discharge energy density at 95% efficiency by 105%. Moreover, the charge–discharge efficiency at 400 MV/m after accelerated aging improves by 81%. Notably, prototype capacitors fabricated using roll‐to‐roll‐coated PEI films demonstrated at least a 45‐fold enhancement in lifetime at both room temperature and 150°C, under an electric field of 180 MV/m—a design field previously only attained by BOPP at temperatures below 60°C, demonstrating the scalability and reliability of this nanocoating strategy at engineering‐relevant scales.

## 2 Results

### 2.1 Thermal‐Electrical Dual Confinements Within Multilaminate Nanocoating

The nanocoating was deposited using a customized roll‐to‐roll continuous coating system (Figure S1). A uniformly exfoliated PVA/MMT dispersion (see Methods) was delivered onto the moving substrate via a motor‐controlled pump and metered by a doctor blade to ensure uniform film application. The coated film was then heated to 130°C, during which gravity‐directed, evaporation‐induced self‐assembly of MMT nanosheets and concurrent cross‐linking produced a highly oriented organic‐inorganic multilaminate coating (∼179 nm thick) at a coating speed of 0.49 m min^−1^. Multiple coating cycles are readily enabled if required. Cross‐sectional transmission electron microscopy (TEM) confirms the well‐ordered multilaminate architecture (Figure 1b), while X‐ray diffraction (XRD) reveals an interlayer spacing of ∼2.97 nm between adjacent MMT nanosheets (Figure S2).

The self‐assembled multilaminate organic‐inorganic nanostructure imposes strong *dual confinements* within the surface barrier coating, markedly enhancing the thermal and insulation properties while synergistically suppressing hot‑carrier injection and transport. First, spatial *nanoconfinement* imposed by the inorganic MMT layer on the organic PVA layer suppresses directly the mobility of PVA chains. Such confinement‐induced deviations from polymer bulk behavior have been widely observed across various polymer‐inorganic systems and are largely associated with attractive interfacial interactions that strongly restrict polymer‐chain dynamics [39, 47, 48]. As illustrated in Figure 1b, abundant hydroxyl groups of PVA, together with the incorporated crosslinking agents, form extensive hydrogen bonding and covalent binding [15, 49, 50] across the organic‐inorganic interfaces, generating strong interfacial attractions [39, 40, 47] that confine PVA chains in ∼2 nm thick layers between adjacent MMT surfaces. This nanoscale confinement preserves high structural coherence throughout the multilaminate coating, further reinforced by interchain crosslinking (Figure 1b), thereby transforming effectively the confined PVA layers into a quasi‐immobilized matrix.

To elucidate the restriction of chain segmental motion induced by nanoconfinement, broadband dielectric spectroscopy (BDS) was performed on neat PVA films (with the same crosslinking ratio) and on PVA/MMT coating layers (see details in Supporting Information). As inorganic materials do not exhibit an *α*‐relaxation process, the segmental dynamics of PVA in the bulk and nanoconfined states can be directly compared by analyzing their *α* relaxations. As shown in Figure 1c and Figures S3–S6, the permittivity dispersion associated with *α* relaxation is substantially more pronounced in the neat PVA than in the PVA/MMT coating. The *α*‐relaxation peak frequencies (*f*_max_) were extracted using the Kohlrausch‐Williams‐Watts (KWW) model, and their temperature dependence was fitted by the Vogel‐Fulcher‐Tammann (VFT) relation (Figures S7–S10 and Table S1). As shown in Figure 1d, the relaxation rate of nanoconfined PVA, characterized by *f*_max_, is nearly an order of magnitude lower than that of bulk‐state PVA at elevated temperatures, confirming strongly suppressed segmental motion. Consistently, differential scanning calorimetry (DSC) results (Figure 1c and Figure S11) show a pronounced glass transition in neat PVA, whereas the glass transition is essentially absent in the PVA/MMT coating layer, indicating that PVA chain segments confined between MMT nanosheets exist in a highly immobilized state [51]. Moreover, atomic force microscope (AFM) based nanoindentation reveals that the PVA/MMT coating layer exhibits a Young's modulus of 34.0 GPa, over an order of magnitude higher than of neat PVA (2.4 GPa) (Figure 1e and Figure S12). While the incorporation of 2D MMT nanosheets is expected to provide the dominant contribution to this enhancement, polymers with restricted chain mobility are also known to exhibit increased stiffness [48, 52]. It has been reported that confinement from a single attractive inorganic‐organic interface can increase the elastic modulus of the polymer by nearly 50% within tens of nm from the interface [48]. Given the numerous interfaces in multilaminate coating and the ∼2 nm thickness of the confined PVA, nanoconfinement is also expected to increase its stiffness and thereby contribute to the overall enhancement in the modulus of the coating.

Beyond modifying mechanical and thermal properties, nanoconfinement of PVA chains plays a critical role in impeding carrier transport within the PVA layers. In polymers subjected to high electric fields, hot‐carrier energy relaxation is governed by optical and acoustic phonon scattering [44, 45], originating from atomic vibrations and segmental molecular motions, respectively [8]. Nanoconfinement restricts the segmental motion of PVA chains and increases their stiffness. This stiffening shifts the vibrational density of states (VDOS) toward higher and broader frequency ranges [53], thus significantly enhancing acoustic‐phonon scattering of hot carriers. At relatively low electric fields, charge transport in polymers is generally governed by thermally activated hopping between localized states in the band tail [12, 54, 55, 56]. At elevated temperatures, intensified thermally induced chain motions lead to pronounced temporal and spatial fluctuations of these localized states [12, 37], which can facilitate carrier transport by increasing tunnelling (hopping) probabilities between nearby unoccupied states [37, 56]. In the present nanocoating, nanoconfinement imposes strong steric hindrance on PVA chain motion, thereby suppressing such fluctuations and effectively inhibiting hopping transport within the organic layer.

Most importantly, besides spatial confinement, electronic confinement arising from anisotropic carrier localization in MMT nanosheets further suppresses *out‐of‐plane* carrier transport, forming a nanometric HV *in‐plane* “rectifier”. To gain atomic‐scale insight into this process, we performed NAQMD simulation on an orthorhombic supercell composed of three MMT 2D nanosheets with inherently anisotropic electronic band structures (the inset of Figure 1f, Figure S13a). This *time‐dependent density‐functional‐theory* (TD‐DFT)‐based approach explicitly captures vibronic coupling, whereby the occupation and transition of electronically excited states are governed by nuclear configurations and vibrational motions [57, 58]. In the simulations, multiple hot electron‐hole pairs were initially excited in the conduction and valence bands. Under an external electric field applied perpendicular to the nanosheets, electrons and holes migrate in opposite directions and undergo nonadiabatic transitions. As shown in the insets of Figure 1g and h and Figure S13b, the spatial distributions of both electrons and holes along the *z* axis are strongly localized near specific atomic centers (e.g., Si─O tetrahedra and Al─O octahedra), whereas their distributions are comparatively delocalized in the *x*‐*y* plane. We further quantified this anisotropy by computing the directional spread functional *σ*_i_^2^, a quantity commonly used in the construction of the Wannier functions, for electron and hole propagating within bulk MMT nanosheets over 1200 fs following excitation. Here, *σ*_i_^2^ is defined as ⟨*ϕ*|*A_i_* ^2^|*ϕ*⟩ − (⟨*ϕ*|*A_i_* |*ϕ*⟩)^2^ + ⟨*ϕ*|*B_i_* ^2^|*ϕ*⟩ − (⟨*ϕ*|*B_i_* |*ϕ*⟩)^2^, where *ϕ* is the carrier wavefunction, and *A*_i_ and *B*_i_ are self‐adjoint operators sin(2π*r_i_*/*L_i_*) and cos(2π*r_i_*/*L_i_*), respectively, under periodic conditions [59]. Statistical analysis (Figure 1g,h) shows that *σ_x_* ^2^ and *σ_y_* ^2^ are close to unity, whereas *σ_z_* ^2^ approaches 0, quantitatively confirming strong anisotropic localization. This behavior reflects the inherently anisotropic electronic structure of 2D MMT nanosheets: the in‐plane covalently bonded lattice supports band‐like transport in the x‐y plane, while weak interlayer coupling—stemming from limited overlap of out‐of‐plane orbitals [60, 61] suppresses carrier transport along the field direction. Consequently, hot‐carrier transport along the electric field is effectively impeded, and carriers are redirected into non‐destructive lateral dissipation pathways, forming a nanometric HV in‐plane rectifier. The simulations further reveal efficient carrier energy relaxation mediated by strong phonon scattering; as shown in Figure 1f, the excitation energy of the *N*‐electron system decays rapidly within several hundred femtoseconds. This ultrafast thermalization within the multilaminate nanostructure clearly demonstrates the *out‐of‐plane “rectification”* and the formation of effective barriers that suppresses hot‐carrier injection into polymer films under combined high‐temperature and high‐electric‐field conditions.

Notably, the synergistic action of redundant through‐thickness barriers and *out‐of‐plane “rectification”* provides the physical basis for the intrinsic defect tolerance of the multilaminate coating. Specifically, exfoliated MMT nanosheets have irregular shapes, therefore, a single self‐assembled MMT layer cannot form perfectly continuous and gap‐free coverage, leaving local discontinuities that act as *defects* within the layer. However, these local gaps in different layers are spatially staggered and can be covered by neighboring MMT layers, therefore preserving effective resistance to through‐thickness carrier injection. Although defects in several adjacent layers may partially overlap and form a relatively continuous through‐plane pathway (i.e., multi‐layer defects), the presence of only a few MMT layers along this pathway can interrupt and redirect the carriers laterally toward other regions. The hundreds of stacked layers further provide substantial redundancy for this mechanism. In this sense, the impact of both single‐ and multi‐layer defects can be effectively mitigated through these synergistic effects. Such defect tolerance is more difficult to achieve in a single‐layer homogeneous coating (Figure 1a), which lacks through‐thickness redundancy and *out‐of‐plane “rectification”*. Once a local defect appears, it acts as a weak region that can intensify the local electric field, promote concentrated injection, and thus facilitate further conduction pathway development.

### 2.2 Enhanced High‐Temperature Insulation Performance of Polymer Films

The dual‐confinement nanocoating significantly improves the insulation performance of heat‐resistant polymer films under concurrent high electric fields and elevated temperatures. To quantify these improvements, the present coating was self‐assembled on a roll of PEI films. We measured the pre‐breakdown conduction current integral (*Q*) of coated and uncoated PEI films as a function of electric field (*E*) at room temperature and 150°C. As shown in Figure 2a, the nanocoating not only suppresses the conduction current but also effectively limits its nonlinear increase at high electric fields. Furthermore, as illustrated in Figure 2b,c, the hopping distance of the coated film at 150°C is reduced by 46%, with a comparable reduction observed at room temperature. These results confirm that the nanocoating both impedes carrier injection and promotes efficient relaxation of injected carriers. Consequently, the Weibull characteristic breakdown strength of polymer film over an area of 1 cm^2^ is significantly enhanced at room temperature (Figure 2d) and improved by 11% at 150°C, increasing from 450 MV/m to 501 MV/m (Figure 2e). In addition, long‐term aging‐induced failures are substantially suppressed. As plotted in Figure 2f, under various electric fields, the time‐to‐breakdown of the coated film is nearly an order of magnitude longer than that of the uncoated film.

**FIGURE 2:**
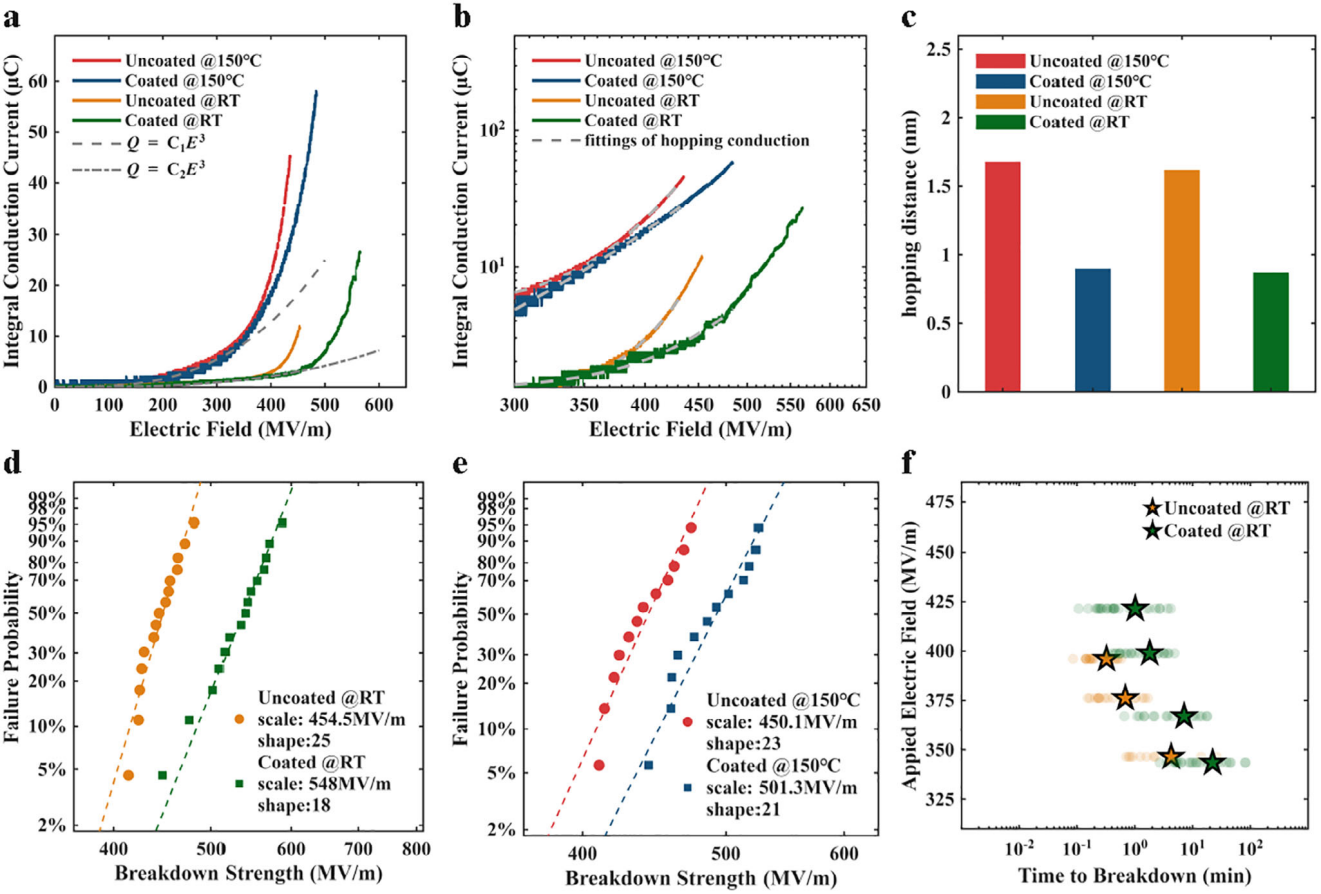
Significant enhancement of high‐temperature high‐field insulation performance of polymer films due to nanocoating. (a) high‐field conduction current integral of PEI films at room temperature and 150°C, and the slope of Q‐E curve exceeds E^3^ after a certain electric field; (b) conduction current integral in log scale and segmental fittings of hopping conduction (details in Supporting Information and Table S2); (c) hopping distances of uncoated and coated films at room temperature and 150°C; Weibull‐fit breakdown strength of uncoated and coated films at (d) room temperature and (e) 150°C; (f) lifetimes of uncoated and coated films under high electric fields. Each circular data point (light orange or light green) represents the time‐to‐breakdown of the film in a single test under a specific electric field, and each star point represents the Weibull characteristic lifetime at the same electric field.

The enhanced insulation performance further translates into improved charge–discharge efficiency and energy‐storage capability under harsh operating conditions. Electric displacement‐electric field (*D*‐*E*) loop measurements show that the coated PEI films achieve higher efficiency at both room temperature and 150°C (Figure 3a and Figure S16). At 150°C, the maximum discharge energy density attained at efficiencies above 95% is increased by 105% (Figure 3b). Large‐area *D*‐*E* loops measurements, conducted using 1‐cm‐diameter circular electrodes at different sections of the film, demonstrate that the nanocoating consistently improves the efficiency by 57% under extreme combined stresses of 400 MV/m and 150°C (Figure 3d,e). Furthermore, large‐area accelerated aging tests were performed under a bipolar sinusoidal waveform (100 Hz, ± 300 MV/m peak‐to‐peak) at 150°C. After 2000 cycles of bipolar HV aging, the preserved efficiency for nanoclay coated PEI tested under harsh conditions (400 MV/m and 150°C) is 81% higher than neat PEI (Figure 3c and Figure S17), demonstrating the long‐term reliability of the nanocoating under alternating electrical stresses at elevated temperatures.

**FIGURE 3:**
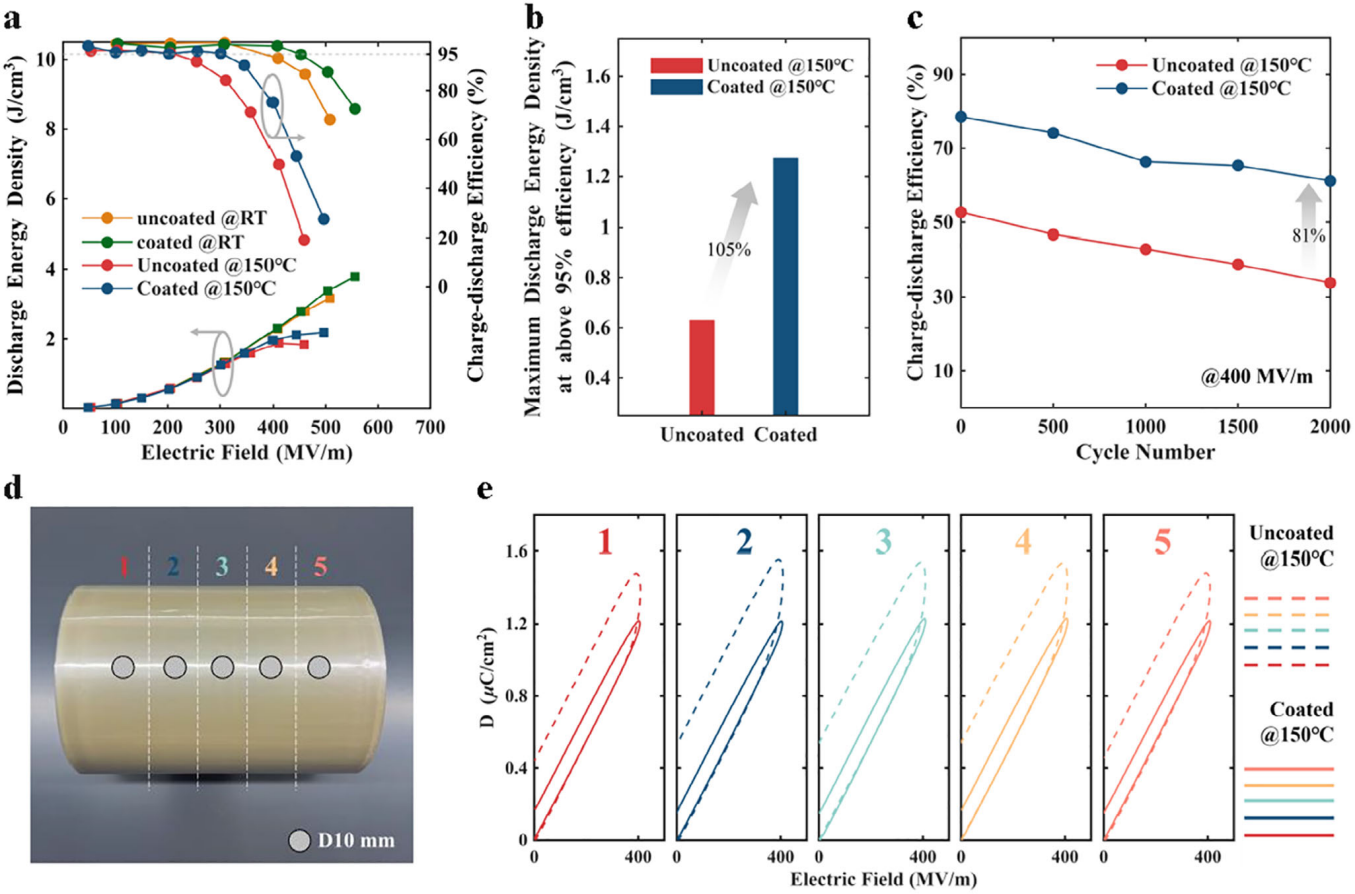
Improved charge–discharge efficiency and energy‐storage capability of polymer films under harsh conditions. (a) discharge energy density and efficiency of uncoated and coating films at room temperature and 150°C; (b) improvement of maximum discharge energy density at above 95% efficiency; (c) efficiency of uncoated and coating films at 150°C and 400 MV/m, which were aged under bipolar sinusoidal HV waveforms; (d) different test region on the roll of coated film; (e) large‐area D‐E loop results tested at different regions shown in d).

### 2.3 Film‐and‐Foil Capacitors With Substantially Extended Operating Limits

To further demonstrate the scalability of the roll‐to‐roll self‐assembled nanocoating, a 40‐m roll of coated PEI film (Figure 4a) was used, for the first time, to fabricate film‐and‐foil capacitors. As illustrated in Figure 4b, the coated PEI film and aluminum foil were wounded around a 3‐mm‐diameter core with a 2‐mm clearing edge. The wound rolls were vacuum evacuated and encapsulated with Kapton film. Subsequently, both ends of capacitors were plasma‐spray metalized, and electrical leads were soldered for testing (Figure 4c and Figure S18). For comparison, capacitors based on uncoated PEI film and aluminum foil were fabricated using the same procedure as a control group.

**FIGURE 4:**
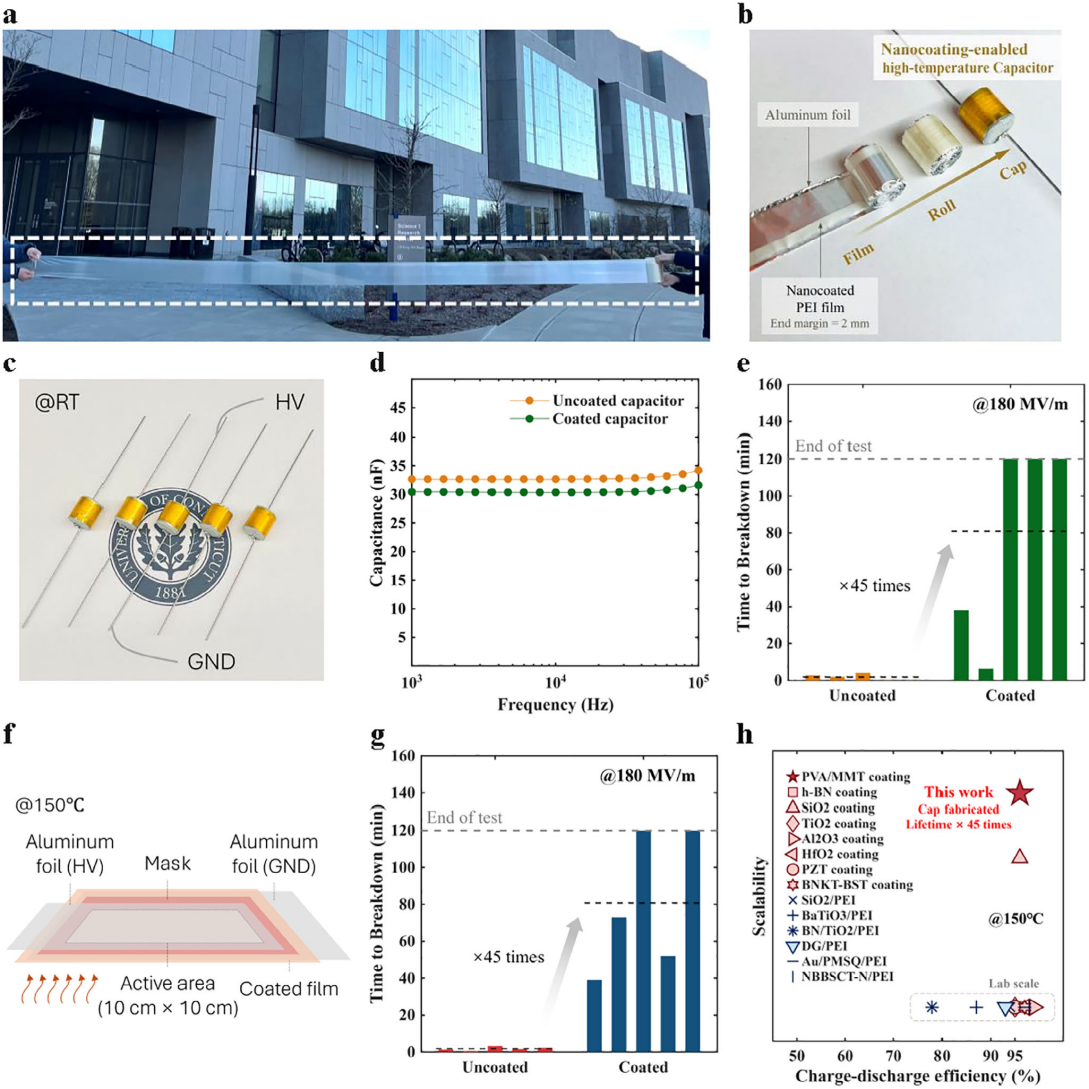
Roll‐to‐roll‐coated capacitors with extended lifecycle. (a) a 40‐m roll of coated PEI film; (b) schematic of fabrication process of film‐and‐foil capacitors; (c) coated PEI film‐and‐aluminum foil capacitors; (d) capacitances of coated and uncoated capacitors at different frequencies; (e) lifetimes of coated and uncoated capacitors at room temperature under 180 MV/m electric field (details in Supporting Information); (f) prototype parallel‐plate capacitors using coated and uncoated films; (g) lifetimes of coated and uncoated prototype capacitors at 150°C under 180 MV/m; (h) comparison of the scalability and efficiency of this work with previously reported coatings on PEI films and PEI‐based composites/nanocomposites (Table S3) at 150°C and 300 MV/m.

As shown in Figure 4d, the capacitances of the uncoated and coated capacitors are 32.6 and 30.5 nF, respectively. The slight reduction in capacitance after coating arises from the increased film thickness, while the nanocoating has no observable effect on the frequency dependence of capacitance. Accelerated aging tests under a direct‐current electric field were conducted to evaluate the reliability of the nanocoating under high‐field stress. As summarized in Figure 4e, the nanocoating extends the average time‐to‐breakdown of these capacitors by at least 45‐fold at an electric field of 180 MV/m. At 150°C, the average time‐to‐breakdown of the prototype capacitors (Figure 4f) is similarly increased by at least 45‐fold (Figure 4g). These results further demonstrate the capability of the present nanocoating in enhancing the operating electric field of the heat‐resistant polymer film to the similar level of BOPP operating below 60°C. Furthermore, Figure 4h compares the scalability and the efficiency of this work with state‑of‑the‑art coatings on PEI films and PEI‑based composites or nanocomposites. The scalability is evaluated by the film area (in m^2^) reported in the literature. For approximately A4‐sized film, the scalability value was normalized to 1 for comparison. Owing to the defect‐tolerant dual‐confinement design and roll‐to‐roll self‐assembly process, the present nanocoating simultaneously enhances the efficiency of heat‐resistant polymer films under extreme electric and thermal stresses while demonstrating excellent manufacturing readiness and robustness for large‐scale applications.

## 3 Conclusions

In this work, we introduce a roll‐to‐roll self‐assembled multilaminate nanocoating to significantly enhance the performance of high‐temperature polymer film capacitor at an engineering‐relevant scale. The roll‐to‐roll process enables well‐controlled self‐assembly of PVA/MMT multilayers, allowing high‐throughput deposition of uniform nanocoatings. Within the defect‐tolerant multilaminate architecture, PVA chains are nanoconfined, and their hindered chain motion forms a quasi‐immobilized matrix with enhanced thermal stability and suppressed carrier transport. NAQMD simulations further reveal that hot carriers undergo anisotropic localization and rapid relaxation within the two‐dimensional nanocoating, forming an effective nanometric in‐plane hot‐carrier rectifier. Together, these effects substantially improve the insulation performance of polymer films at 150°C, including reduced conduction (hopping distance decreased by 46%), enhanced breakdown strength (increased by 11%), increased discharge energy density (by 105%), and improved charge–discharge efficiency (by 81%).

Notably, roll‐to‐roll coated PEI films were, **for the first time**, fabricated into film‐and‐foil capacitors explicitly designed to operate at 150°C under an electric field of 180 MV/m—a design field previously achieved only by BOPP at temperatures below 60°C. Under this operating field at both room temperature and 150°C, the nanocoating delivers at least a 45‐fold enhancement in the measured lifetime of heat‐resistant polymer films, demonstrating exceptional scalability, reliability, and technological readiness for next‑generation high‑temperature, high‐energy‐density power capacitors.

## 4 Methods

### 4.1 Materials

To prepare the nanocomposite coating dispersion, PVA (POVAL 22–88 from Kuraray America, Inc.) was first dissolved in deionized (DI) water with the assistance of heating until a homogenous solution was formed. PVA was selected through binder screening because of its favorable compatibility with the aqueous self‐assembly process, whereas several intrinsically more thermally stable binders showed insufficient solubility. In addition, the hydroxyl‐rich PVA chains can readily adsorb onto the hydrophilic surfaces of MMT nanosheet, which are critical for enabling the flow‐induced alignment and co‐assembly of MMT nanosheets. Subsequently, MMT (Cloisite Na^+^ MMT from BYK USA Inc.) was dispersed in DI water and ultrasonicated (Branson M880, 280 W, 40 kHz) in order to exfoliate its layered structure into individual high‐aspect ratio nanosheets. The aqueous PVA solution was combined with the exfoliated MMT dispersion while stirring to form a solution with a total solid content of 1.5% (w/w), with a 1:1 ratio of PVA to MMT. After stirring, the PVA/MMT mixture was ultrasonicated again to ensure the formation of a homogeneous PVA/MMT dispersion. Finally, a crosslinking agent, glutaraldehyde (GA, 50% from Thermo Scientific), was added to the mixture, where the total mole ratio of crosslinker to hydroxyl groups on PVA was 1:20. The prepared coating was then applied to PEI films (UTF120, 10 µm in thickness) by using a modified continuous coating machine (TOB‐TBJ‐180C, TOB New Energy). A total of 50‐meter‐long and 6‐inch‐wide film was coated, of which 40‐meter film was used for capacitor fabrication. All film capacitors are manufactured by Electronic Concepts, Inc. USA.

### 4.2 Characterization

*XRD* patterns were recorded on a Bruker D2 diffractometer using a graphite monochromator with Cu K_α_ radiation. The coated PEI films were embedded in epoxy and microtomed into thin slices using a Reichert‐Jung Ultracut E ultramicrotome. *TEM* images were captured by a Tecnai T‐12 transmission electron microscopy. *DSC* was performed on a TA Instruments DSC Q‐100 differential scanning calorimeter, using a temperature ramp of 10°C/min. *BDS* was conducted in *Navel Research Lab* within N_2_ environment, and all the data were obtained during third cooling cycle. The *AFM based nanoindentation* measurements were carried out in the nanomechanical mapping mode on Asylum Research Cypher VRS AFM using the Bruker (TESPA‐V2) and Oxford Instruments (HQ‐150‐Au) cantilevers (more details in Supporting Information).

### 4.3 High Electric Field Tests

*High field D‐E loop measurement* was employed using a modified Sawyer‐Tower polarization loop tester with a unipolar positive half sinusoidal wave of 100 Hz. The measurement system consisted of a Trek Model 10/40 10 kV high voltage amplifier and an OPA541 operational amplifier‐based current‐to‐voltage converter. Gold/palladium electrodes are coated on both sides of the film using the sputter coating method. *High field conduction current integral* was measured with a specially designed measurement system which can dynamically cancel the capacitive component of the current [62]. A small sinusoidal modulation signal was superimposed onto the voltage ramp to track the transient capacitive response. Through a negative feedback loop with a dual‐phase lock‐in amplifier, the capacitive current was continuously canceled via adaptive gain control, enabling detection of resistive currents with a resolution down to 10 ppm. *Breakdown and time‐to‐breakdown measurements* were detailed in Figures S14 and S15.

## Author Contributions

**Ning Guo**: investigation, writing – original draft, validation, visualization, writing – review and editing, formal analysis, data curation, software. **Marina Dabaghian**: investigation, validation, writing – review and editing, formal analysis. **Jierui Zhou**: investigation, writing – review and editing. **Josh N. Bodin**: investigation, writing – review and editing, formal analysis, validation. **Chong Xiang**: investigation, writing – review and editing, formal analysis, validation, data curation. **Fei Wang**: investigation, visualization, validation, writing – review and editing, data curation. **Antigoni Konstantinou**: formal analysis, writing – review and editing. **Dingxin Wei**: investigation, writing – review and editing, validation. **Yifei Wang**: writing – review and editing. **Chao Wu**: writing – review and editing. **Riccardo Casalini**: writing – review and editing, formal analysis, investigation. **Bryan Huey**: writing – review and editing, supervision, conceptualization. **Priya Vashishta**: conceptualization, visualization, writing – review and editing, methodology, software, resources. **Luyi Sun**: conceptualization, writing – review and editing, supervision, methodology. **Yang Cao**: conceptualization, funding acquisition, investigation, writing – original draft, writing – review and editing, methodology, formal analysis, project administration, supervision, resources.

## Conflicts of Interest

The authors declare no conflicts of interest.

## Supporting information




**Supporting File**: advs78275‐sup‐0001‐SuppMat.docx.

## Data Availability

The data that support the findings of this study are available from the corresponding author upon reasonable request.
