# Supplementary material for: Interface Rectification via Roll‐to‐Roll Dual‐Confinement Nanocoatings for High‐Temperature Polymer Capacitors
Source: Adv Sci (Weinh). 2026 Oct 14:e78275. Online ahead of print. doi: 10.1002/advs.78275 (PMC13669228; doi:10.1002/advs.78275)
Supplement: Supplementary file 1 [file ADVS-9999-e78275-s001.docx]

Supplementary Information

Interface rectification via roll-to-roll dual-confinement nanocoatings for high-temperature polymer capacitors

Ning Guo^a, b †^, Marina Dabaghian^c, d, †^, Jierui Zhou^e^, Josh N. Bodin^c, d^, Chong Xiang^c, d^, Fei Wang^f^, Antigoni Konstantinou^a^, Dingxin Wei^a, b^, Yifei Wang^a^, Chao Wu^a^, Riccardo Casalini^g^, Bryan Huey^h^, Priya Vashishta^i^, Luyi Sun^c, d, *^, and Yang Cao^a, b, *^

^a^ Electrical Insulation Research Center, Institute of Materials Science, University of Connecticut, Storrs, CT 06269, USA

^b^ Department of Electrical and Computer Engineering, University of Connecticut, Storrs, CT 06269, USA

^c^ Department of Chemical and Biomolecular Engineering, University of Connecticut, Storrs, CT 06269, USA

^d^ Polymer Program, Institute of Materials Science, University of Connecticut, Storrs, CT 06269, USA

^e^ GE Vernova, Advanced Research Center, Niskayuna, NY 12309, USA

^f^ Department of Materials Science & Engineering, University of Connecticut, Storrs, CT 06269, USA

^g^ Chemistry Division, Naval Research Laboratory, Washington DC 20375, USA

^h^ School of Materials Engineering, Purdue University, West Lafayette, IN 47907, USA

^i^ Collaboratory for Advanced Computing and Simulations, University of Southern California, Los Angeles, CA 90089, USA

**Content**

[Supplementary Fig. 1	3](#_Toc240539972)

[Supplementary Fig. 2	3](#_Toc240539973)

[Supplementary Fig. 3	5](#_Toc240539974)

[Supplementary Fig. 4	5](#_Toc240539975)

[Supplementary Fig. 5	6](#_Toc240539976)

[Supplementary Fig. 6	6](#_Toc240539977)

[Supplementary Fig. 7	7](#_Toc240539978)

[Supplementary Fig. 8	7](#_Toc240539979)

[Supplementary Fig. 9	8](#_Toc240539980)

[Supplementary Fig. 10	8](#_Toc240539981)

[Supplementary Fig. 11	9](#_Toc240539982)

[Supplementary Fig. 12	11](#_Toc240539983)

[Supplementary Fig. 13	12](#_Toc240539984)

[Supplementary Fig. 14	14](#_Toc240539985)

[Supplementary Fig. 15	14](#_Toc240539986)

[Supplementary Fig. 16	15](#_Toc240539987)

[Supplementary Fig. 17	16](#_Toc240539988)

[Supplementary Fig. 18	18](#_Toc240539989)

[Supplementary Table 1.	4](#_Toc240897535)

[Supplementary Table 2.	13](#_Toc240897536)

[Supplementary Table 3.	19](#_Toc240897537)

1. **Roll-to-roll self-assembled nanocoating** **setup**

Supplementary Fig. 1 Roll-to-roll self-assembled nanocoating setup.

Supplementary Fig. 2 X-ray diffraction (XRD) measurements of the coatings deposited via roll-to-roll coating process present in this work and via manually dip coating process^1^.

2. **Broadband Dielectric Spectroscopy (BDS) and Differential Scanning Calorimetry (DSC)**

To prepare neat PVA films, a 1.5wt% aqueous PVA solution was first prepared. The crosslinking agent was then added to the solution, where the total mole ratio of crosslinker to hydroxyl groups on PVA was 1:20. Subsequently, the solution was cast on a glass plate, metered by doctor blade, and then dried under 80 ℃ for 2 hours. Finally, the PVA film was delaminated from the glass plate and used for both BDS and DSC measurements. For simplicity, the “neat PVA” was used to refer to the PVA film containing the same ratio of crosslinking.

To prepare PVA/MMT coating layers, the same solution detailed in **Method** was prepared first. Then, the self-assembling process was completed on the PET substrate, and the coating layers were delaminated from the substrate. These coating layers were used for DSC measurements. For BDS measurements, the proposed nanocoating was self-assembled on the brass metal sheet. This metal sheet served as an electrode of the coating layer. We then sputter-coated a gold/palladium electrode, 20 mm in diameter, on another side of the coating layer. These supported coating layers are used for BDS measurements. The BDS measurement was conducted in *Navel Research Lab* within N_2_ environment, and all the data were obtained during the third cooling cycle to effectively remove the influence of moisture.

The electrical modulus was calculated based on the dielectric spectroscopy. Kohlrausch-Williams-Watts (KWW) relaxation model^2,3^ and Havriliak-Negami (HN) relaxation model^4^ in the form of electrical modulus were used to extract the peak frequencies (*f*_max_) of different relaxation processes (*α* relaxation, *β* relaxation, electrode polarization). The relationships between the peak frequencies of *α* relaxation and the inverse of temperatures were characterized by Vogel-Fulcher-Tamman (VFT) relation, as shown in equation (1)^4^.

|  | $\text{v}\text{ }\text{=}{\text{ }\text{v}}_{\text{∞}}\text{exp(}\frac{\text{–}\text{D}\text{T}_{\text{0}}}{\text{T}\text{ }\text{–}\text{ }\text{T}_{\text{0}}}\text{)}$ | (1) |
| --- | --- | --- |
|  | log($\text{v}$) = log$\text{v}_{\text{∞}}$ + log(exp($\frac{\text{–}\text{D}\text{T}_{\text{0}}}{\text{T}\text{ }\text{–}\text{ }\text{T}_{\text{0}}}$)) | (2) |
|  | log($\text{v}$) = log$\text{v}_{\text{∞}}$ – 0.4343$\frac{\text{D}\text{T}_{\text{0}}}{\text{T}\text{ }\text{–}{\text{ }\text{T}}_{\text{0}}}$ | (3) |

where *D* is a constant, and *T*_0_ is the Vogel temperature. All fitting parameters for Fig. 1c are summarized below.

Supplementary Table 1. All VFT fitting parameters for Fig. 1c.

|  | log$\text{v}_{\text{∞}}$ | *D* | *T*_0_ |
| --- | --- | --- | --- |
| neat PVA | 12.98 | 15.54 | 226.10 |
| PVA/MMT coating layer | 9.30 | 6.81 | 263.03 |

Supplementary Fig. 3 Dielectric constant of the neat PVA film.

Supplementary Fig. 4 Dielectric loss of the neat PVA film.

Supplementary Fig. 5 Dielectric constant of the PVA/MMT coating layer.

Supplementary Fig. 6 Dielectric loss of the PVA/MMT coating layer.

Supplementary Fig. 7 Imagery part of electrical modulus of the neat PVA film.

Supplementary Fig. 8 Imagery part of electrical modulus of the PVA/MMT coating layer.

Supplementary Fig. 9 Example fitting (80 ℃ spectra) of *α* process of the neat PVA film using KWW relaxation model. The electrode polarization and *β* process were fitted using HN relaxation model.

Supplementary Fig. 10 Example fitting (150 ℃ spectra) of *α* process of the PVA/MMT coating layer using KWW relaxation model. The electrode polarization and *β* process were fitted using HN and KWW relaxation model, respectively.

Supplementary Fig. 11 DSC results of the neat PVA and the PVA/MMT coating layer (the second heating cycle).

3. **Atomic Force Microscope (AFM) based nanoindentation**

The AFM based nanoindentation measurements were carried out in the nanomechanical mapping mode on Asylum Research Cypher VRS AFM. Since the stiffness of the neat PVA and the PVA/MMT coating layer have a large difference, we used two cantilevers with different spring constants. For each sample, at least three rounds of measurements were performed, and one representative image was given here for each sample. Supplementary Fig. 12a and b illustrate the schematic of AFM-based nanoindentation.

- **Neat PVA film:** a silicon cantilever with a calibrated spring constant of 5.4 N/m (HQ-150-Au, Oxford Instruments) was used in this measurement, and the actual spring constant was measured by a thermal tuning method. Force curves were collected over randomly selected surface areas of 4 μm × 4 μm at an elastic modulus resolution of 125 × 125 pixels (1024 data points). The sample preparation was the same as that used in DSC measurements.
- **PVA/MMT coating layer**: a silicon cantilever with a calibrated spring constant of 23.5 N/m (TESPA-V2, Bruker) was used in this measurement, and the actual spring constant was measured by a thermal tuning method. Force curves were collected over randomly selected surface areas of 4 μm × 4 μm at an elastic modulus resolution of 80 × 80 pixels (2500 data points). The sample preparation was the same as that used in DSC measurements.

The classic Hertz model was used to derive the local elastic modulus from indentation loading curves (force-depth loading curve).

$$\text{F}_{\text{Hertz}}\text{=}\frac{\text{4}}{\text{3}}\text{E}^{\text{*}}\sqrt{\text{R}^{\text{*}}\text{δ}^{\text{3}}}$$

(4)

$$\frac{\text{1}}{\text{E}^{\text{*}}}\text{=}\frac{\left( \text{1}\text{ }\text{–}\text{ }\text{υ}_{\text{tip}}^{\text{2}} \right)}{\text{E}_{\text{tip}}}\text{+}\frac{\left( \text{1}\text{ }\text{–}\text{ }\text{υ}_{\text{sample}}^{\text{2}} \right)}{\text{E}_{\text{sample}}}$$

(5)

$$\frac{\text{1}}{\text{R}^{\text{*}}}\text{=}\frac{\text{1}}{\text{r}_{\text{tip}}}\text{+}\frac{\text{1}}{\text{r}_{\text{sample}}}$$

(6)

where *E*^*^ is the reduced modulus which can be related to film elastic modulus, *ν*_sample_ is the Poisson’s ratio of the film. For the polymer film samples, we used a Poisson’s ratio of 0.33, measured from tensile test simulations of the bulk system with periodic boundary conditions applied in all directions. As mentioned above, the tip used in the simulations is a completely rigid repulsive indenter (i.e., *E*_tip_ = ∞).

| **a**   | **b**   |
| --- | --- |
|  |  |
| **c**   | **d**   |
| **e**   | **f**   |

Supplementary Fig. 12 Schematic illustrating AFM-based nanoindentation on a) the neat PVA and b) the PVA/MMT nanocoating; elastic modulus maps of c) neat PVA and d) PVA/MMT coating layer; elastic modulus distribution of e) neat PVA (2.4 ± 0.2 GPa) and f) PVA/MMT coating layer (34.04 ± 7.3 GPa).

4. **Nonadiabatic Quantum Molecular Dynamics (NAQMD) simulation**

TD‑DFT-based NAQMD simulations were conducted on the orthorhombic supercell of three nanosheets under periodic boundary conditions using QXMD software with NVT ensemble. Nonadiabatic transitions between electronic states were determined using surface hopping method^5^. The electronic structures were calculated based on the projector augmented-wave (PAW) method^6^ using a plane-wave basis. Projector functions were defined for H (1s), O (2s, 2p), and Na, Al, and Si (3s, 3p, 3d) states. The cutoffs were set to 35 Ry for wavefunctions and 300 Ry for densities. Brillouin zone sampling was carried out using the Γ-point. Exchange-correlation functional was treated using the Perdew-Burke-Ernzerhof (PBE) generalized gradient approximation, and Van der Walls interactions were added with the dispersion-corrected density functional theory approach.

The average electronic excitation energy of the *N*‑electron system is described as in equation (7):

$$E_{exc}\left( t \right)=\frac{\sum_{i} f_{i}\left( t \right)\in_{i}\left( t \right) \theta(\in_{i}(t))-\sum_{i} \left( 1-f_{i}\left( t \right) \right)\in_{i}\left( t \right) \theta(-\in_{i}(t))}{\sum_{i} f_{i}\left( t \right)\in_{i}\left( t \right) \theta(\in_{i}(t))+\sum_{i} \left( 1-f_{i}\left( t \right) \right)\in_{i}\left( t \right) \theta(-\in_{i}(t))}$$

(7)

where *f_i_*(*t*) denotes the occupation of the *i*^th^ electronic state at time *t*, $\text{∈}$*_i_*(*t*) is the corresponding Kohn–Sham energy, and *θ* represents the Heaviside function.

|  |  |
| --- | --- |

Supplementary Fig. 13 a) Anisotropic carrier localization within MMT nanosheets and b) NAQMD simulation. Electrons and holes are localized along the z axis, whereas they are comparatively extended (uniform) across the x-y plane. This is mainly due to the anisotropic electronic structure of 2D MMT nanosheets: the in plane covalently bonded lattice supports band-like transport in the x-y plane, while the weak interlayer coupling due to limited overlap of out of plane orbitals leads to localization along the z axis.

5. **High field characterization**

The hopping conduction model^7^ is used to evaluate the hopping distance of coated and uncoated samples from the high-field conduction current integral. Derived from equation (8) and (9), the hopping conduction current integral has a form shown in equation (10).

$$J_{H}=2neRve^{-\frac{W}{kT}}\sinh\left( \frac{ReE}{2kT} \right)$$

(8)

$$J=\frac{dQ}{dt}=\frac{e_{0}}{d}\frac{dQ}{dE}$$

(9)

$$Q=A\cosh\left( BE \right)+C$$

(10)

$$R=\frac{2BkT}{e}$$

(11)

where *R* is the hopping distance, *v* is the attempt-to-escape frequency, *W* is the barrier height, and *k* is the Boltzmann constant. *A*, *B* and *C* are three parameters to be optimized to fit the measured conduction current integral. Once the fitting is completed, hopping distance can be derived from equation (11), where *T* is the temperature and *e* is the elementary charge. In Fig. 2b, all current integrals are fitted between 300 MV/m and 435 MV/m except “coated @RT”, which is fitted between 300 MV/m and 485 MV/m to achieve higher *r*^2^ value.

Supplementary Table 2. *r*^2^ value of segmental fittings of hopping conduction.

| **Sample name** | ***r*^2^ value** |
| --- | --- |
| uncoated @RT | >0.99 |
| coated @RT | 0.98 |
| uncoated @150 ℃ | >0.99 |
| coated @150 ℃ | >0.99 |

Supplementary Fig. 14 Breakdown test setup. Metallized polymer films were employed as high voltage (HV) and ground (GND) electrodes, sandwiching the sample and a mask in between. The mask has a 1 cm by 1 cm window as the test area. A ramping voltage (250 V/s) was applied until breakdown. All samples were stored in a vacuum oven for at least 1 week and dried at 60°C for 10 mins before all dielectric tests, and the tests are conducted at room temperature and 150 ℃, respectively.

Supplementary Fig. 15 The long-term aging test setup. The metallized polypropylene (PP) film served as the HV and GND electrode. The polymer film under test was placed in between in a roll configuration. A mask with an active window of 1 cm × 1 cm was inserted below the sample and HV electrode. A constant voltage was applied until electrical breakdown occurred, and the time-to-breakdown was recorded automatically. Upon breakdown, the resulting ground potential rise triggered the control system, which activated the system to replace the failed electrode and sample with fresh ones.

| **a**   | **b**   |
| --- | --- |
| **c**   | **d**   |

Supplementary Fig. 16 *D*-*E* loop results of a) uncoated PEI film at room temperature, b) coated PEI film at room temperature, c) uncoated PEI film at 150 ℃, d) coated PEI film at 150 ℃. 3-mm diameter circular electrode was used during the experiment (Fig. 3a).

| **a** |  | **e** |  |
| --- | --- | --- | --- |
| **b** |  | **f** |  |
| **c** |  | **g** |  |
| **d** |  | **h** |  |

Supplementary Fig. 17 *D*-*E* loop results of large-area (10-mm diameter circular electrode) accelerated aged samples. Uncoated (a-d, corresponding to 500, 1000, 1500, 2000 cycles) and coated (e-h, corresponding to 500, 1000, 1500, 2000 cycles) samples were aged under a bipolar sinusoidal waveform (100 Hz, +/–300 MV/m peak-to-peak) at 150 ℃. The *D*-*E* loop was then conducted at 400 MV/m at 150 ℃. For the 2000-cycle-aged uncoated PEI, the dielectric loss measured with the 10-mm-diameter electrode was too high that a complete *D–E* loop could not be captured within the instrument range. Therefore, a 6-mm-diameter electrode was used to determine the charge-discharge efficiency. Since reducing the stressed area generally improves dielectric performance through size-scaling effects, this electrode substitution is expected to benefit the uncoated sample and thus provides a conservative assessment of the coating effect. In contrast, the 2000-cycle-aged coated PEI remained measurable using the 10-mm-diameter electrode. Even under this conservative comparison, the coated film still exhibited an 81% improvement in charge-discharge efficiency.

6. **PEI capacitor fabrication and testing**

All aluminum-foil-and-film capacitors are manufactured by Electronic Concepts, Inc. USA.

To age the fabricated capacitors, the high voltage output and the ground of the power supply were respectively connected to each lead of the capacitors, and a ramp voltage (50 V/s) was applied until the target electric field was achieved and then the voltage was maintained until the capacitor survives for 120 minutes at the target electric field. If the breakdown occurs, the time-to-breakdown was recorded. Some uncoated capacitors breakdown during the ramping process. The target electric field is selected based on the Weibull mean breakdown strength of uncoated PEI capacitors at room temperature (Supplementary Fig. 18). The aging tests of the prototype parallel-plate capacitors were the same as that of fabricated capacitors.

Supplementary Fig. 18 Weibull fit breakdown strength of uncoated PEI capacitors (63.2% breakdown strength: 188 MV/m, shape parameter: 8.7). The high voltage output and the ground are respectively connected to each lead of the uncoated PEI capacitors, and a ramp voltage (250 V/s) is applied until breakdown occurs at room temperature.

Supplementary Table 3. Discharge efficiency of this work and previously reported coatings on PEI films and PEI-based composites/multilayer-composites at 150 ℃ and 300 MV/m. The comparison is shown further shown in Fig. 4h. While there has been extensive research on PEI—a material holding great potential using as high-temperature capacitor film—most of these efforts remain at the laboratory scale. Very few possesses scalability.

| Dielectric | Discharge efficiency (%) | Literature | Scalability |
| --- | --- | --- | --- |
| PVA/MMT coating | 96 | This work | Capacitor fabricated |
| PEI | 85 | This work | Capacitor fabricated |
| *h*-BN coating | 96 | Azizi et al., *Advanced Materials*, 2017 | Lab scale |
| SiO_2_ coating | 96 | Zhou et al., *Advanced Materials*, 2018 | Roll fabricated |
| TiO_2_ coating | 95 | Cheng et al., *Energy Storage Materials*, 2021 | Lab scale |
| Al_2_O_3_ coating | 99 | Cheng et al., *Energy Storage Materials*, 2021 | Lab scale |
| HfO_2_ coating | 97 | Cheng et al., *Energy Storage Materials*, 2021 | Lab scale |
| PZT coating | 95 | Chi et al., *Ceramics International*, 2023 | Lab scale |
| BNKT-BST coating | 97 | Guo et al., *Advanced Materials*, 2025 | Lab scale |
| SiO_2_/PEI composite | 94 | Sun et al., *Small*, 2022 | Lab scale |
| BaTiO_3_/PEI composite | 87 | Dang et al., *Advanced Functional Materials*, 2024 | Lab scale |
| BN/TiO_2_/PEI multilayer | 78 | Wang et al., *Advanced Materials*, 2021 | Lab scale |
| DG/PEI composite | 93 | Yang et al., *Nature Communications*, 2024 | Lab scale |
| Au/PMSQ/PEI  multilayer | 97 | Chang et al., *Small*, 2024 | Lab scale |
| NBBSCT-N/PEI  multilayer | 98 | Zhao et al., *Nature Communications*, 2025 | Lab scale |

Reference

1.	Ding, F. *et al.* Biomimetic nanocoatings with exceptional mechanical, barrier, and flame-retardant properties from large-scale one-step coassembly. *Sci. Adv.* **3**, (2017).

2.	Bergman, R. General susceptibility functions for relaxations in disordered systems. *J. Appl. Phys.* **88**, 1356–1365 (2000).

3.	Praharaj, S. & Rout, D. Electrical conductivity and modulus study of 0.76Na0.5Bi0.5TiO3-0.2SrTiO3-0.04BaTiO3 ceramic: A near morphotropic phase boundary composition. *Journal of Physics and Chemistry of Solids* **127**, 52–59 (2019).

4.	Kremer, F. & Schönhals, A. *Broadband Dielectric Spectroscopy*. (Springer Science & Business Media, 2002).

5.	Craig, C. F., Duncan, W. R. & Prezhdo, O. V. Trajectory Surface Hopping in the Time-Dependent Kohn-Sham Approach for Electron-Nuclear Dynamics. *Phys. Rev. Lett.* **95**, 163001 (2005).

6.	Blöchl, P. E. Projector augmented-wave method. *Phys. Rev. B* **50**, 17953–17979 (1994).

7.	Mott, N. F. . & Davis, E. A. . *Electronic Processes in Non-Crystalline Materials*. (Oxford University Press, 2012).
